# Dietary Patterns Are Associated with Cognition among Older People with Mild Cognitive Impairment

**DOI:** 10.3390/nu4111542

**Published:** 2012-10-25

**Authors:** Susan J. Torres, Nicola T. Lautenschlager, Naiyana Wattanapenpaiboon, Kathryn R. Greenop, Christopher Beer, Leon Flicker, Helman Alfonso, Caryl A. Nowson

**Affiliations:** 1 Centre for Physical Activity and Nutrition, School of Exercise and Nutrition Sciences, Deakin University, Burwood 3125, Victoria, Australia; Email: naiyana.wattanapenpaiboon@deakin.edu.au (N.W.); caryl.nowson@deakin.edu.au (C.A.N.); 2 Academic Unit for Psychiatry of Old Age, St Vincent’s Health, Department of Psychiatry, University of Melbourne, Melbourne 3052 Australia; Email: nicolatl@unimelb.edu.au; 3 Division of Population Sciences, Telethon Institute for Child Health Research, Centre for Child Health Research, University of Western Australia, Crawley 6009, Western Australia, Australia; Email: kgreenop@ichr.uwa.edu.au; 4 Western Australia Centre for Health and Ageing, Western Australian Institute for Medical Research and Centre for Medical Research, University of Western Australia, Crawley 6009, Western Australia, Australia; Email: christopher.etherton-beer@uwa.edu.au (C.B.); Helman.Alfonso@uwa.edu.au (H.A.); 5 School of Medicine and Pharmacology, University of Western Australia, Western Australia, Crawley 6009, Australia; Email: leon.flicker@uwa.edu.au

**Keywords:** dietary patterns, cognition, mild cognitive impairment, executive function, memory

## Abstract

There has been increasing interest in the influence of diet on cognition in the elderly. This study examined the cross-sectional association between dietary patterns and cognition in a sample of 249 people aged 65–90 years with mild cognitive impairment (MCI). Two dietary patterns; whole and processed food; were identified using factor analysis from a 107-item; self-completed Food Frequency Questionnaire. Logistic regression analyses showed that participants in the highest tertile of the processed food pattern score were more likely to have poorer cognitive functioning; in the lowest tertile of executive function (OR 2.55; 95% CI: 1.08–6.03); as assessed by the Cambridge Cognitive Examination. In a group of older people with MCI; a diet high in processed foods was associated with some level of cognitive impairment.

## 1. Introduction

The world population is aging, with the number of people aged 60 years or over expected to triple over the next 50 years [1]. Cognitive impairment is common among older people. Mild cognitive impairment (MCI) is a frequently used diagnostic concept, defined by objective cognitive impairment in combination with subjective decline but without dementia [2]. Individuals with MCI may have subtle deficits in instrumental activities of daily living, have a higher risk of developing dementia (most commonly Alzheimer’s disease [3]) and have higher rates of mortality [4]. 

There has been increasing interest in the influence of dietary patterns on cognition. Examining dietary patterns enables the analysis of potentially interactive and antagonistic effects of different nutrients. The Mediterranean diet, characterised by a diet high in fish, fruits and vegetables rich in antioxidants, has been associated with a reduction in the risk of Alzheimer disease in an older population [5]. Among an ageing population, a dietary pattern consistent with the Dietary Guidelines for Americans (including fruits, vegetables, whole grains, nuts, fatty fish, and low fat dairy products) was associated with better cognition when compared with a diet that contained few of these foods [6]. When examining the relationship between diet and cognition, it is important to consider the influence of other potentially protective factors, such as physical activity, that may modify the relationship. The many health benefits of physical activity include reductions in fat mass and blood pressure, prevention of coronary heart disease, diabetes and stroke [7] and improvements in cognition [8]. 

The aim of this study was to determine the association between dietary patterns, cognition and physical activity in a cross-sectional sample of older adults with MCI.

## 2. Methods

This study utilised the baseline data from a randomised controlled trial (ANZCTR Number ACTRN12607000321448) examining the effect of an 18-month randomised, double-blind, placebo-controlled clinical trial to establish whether vitamin D supplementation can delay progression of cognitive decline amongst older adults with MCI who have low vitamin D concentrations (serum 25-hydroxy vitamin D level 12.5–70 nmol/L). 

### 2.1. Participant Characteristics

Participants for the study were recruited through media advertisement and announcements, as well as invitation letters to memory clinic patients and participants from previous studies by the research team. 

Participants were eligible for the study if they:

- were 65 years of age or over;
- had subjective memory complaints and cognitive scores below age and gender matched controls (scoring 1.0 SD or more below age- and sex-matched norms on at least one test of the Consortium to Establish a Registry for Alzheimer’s disease (CERAD) battery [9] and Clinical Dementia Rating (CDR) score of 0.5 [10]);
- had Telephone Interview for Cognitive Status-Modified (TICS-M) score of 20 or higher [11];
- were living in community-dwelling settings located in Australia;
- had a score of 24 or higher on the Mini-Mental State Examination [12];
- had relative vitamin D insufficiency (12.5–70 nmol/L); and
- had serum creatinine below 200 µmol/L.

Potential participants were excluded if they:

- had a known diagnosis of dementia;
- had a clinical history of stroke;
- were unstable or had life threatening medical condition;
- had history of schizophrenia;
- had hearing or visual impairments that would preclude assessments;
- had a history of falls with fractures;
- had a history of osteoporosis or medical need for vitamin D supplementation;
- had a history of kidney or bladder stones;
- had a history of drug or alcohol misuse;
- were unable to understand spoken or written English; and
- were unable to attend the visits throughout the study.

All participants provided written informed consent. The study was approved by the Royal Perth Hospital Ethics committee, reference EC 2007/079 in August 2007.

Nine hundred potential participants responded to the recruitment strategies for the randomised controlled trial. Over half were excluded following screening by phone, with 374 attending the screening assessment, and 249 passing the screening assessment and had a sample of blood taken to confirm vitamin D levels. The final sample in this study was 249, with 155 men and 94 women. 

### 2.2. Exposure of Interest: Dietary Patterns

An 107-item self administered food frequency questionnaire (FFQ), adapted from the validated 1995 National Nutrition Survey, assessed usual frequency of intake of food and beverages over the previous 4 weeks [13]. Each item had a choice of 9 frequency categories ranging from “never or less than once per month” to “6 or more times per day”, but did not include any information on serving size. The 107 items of the FFQ were grouped into 33 predefined food groups by adding food items within each group eg Other vegetables included 12 items: vegetable, tomato juice; stir-fried or mixed vegetables; vegetable casserole; pumpkin; sweet potato; carrots; zucchini, eggplant , squash; capsicum; sweetcorn, corn on the cob; mushrooms; celery, cucumber; onion or leeks. Frequency of consumption of the 33 predefined food groups were converted to daily equivalent frequencies and entered into the factor analysis. Dietary patterns were derived from the 33 food groups using factor analysis with factor loadings extracted using the principal component method and varimax rotation using data from the FFQ. Two dietary patterns were identified using multiple criteria: the diagram of eigenvalues, the scree plot, the interpretability of the factors and the percentage of factors explained by the factors (Table 1). The two dietary patterns were confirmed by a statistician. The factor score for each pattern was calculated by summing intakes of all food groups weighted by their factor loadings. Factor loadings represent correlation coefficients between the food groups and particular patterns. The first pattern “whole food” was heavily loaded by a high intake of vegetables, legumes, fruit, whole grain, fish, egg, low fat dairy, nuts and salad dressing. Although salad dressings are typically energy dense, it is likely that it was included in the whole food pattern because it is highly correlated with salad intake. The second pattern, labelled “processed food”, was heavily loaded by a high consumption of processed foods high in saturated fat (desserts, biscuits, fried foods, snacks, high fat takeaway, chocolate and sweets, processed meat and fish), high fat dairy, potatoes, refined grains, sugar beverages and red meat. Each participant received a factor score for the two identified patterns. 

**Table 1:** Factor loadings for the two dietary patterns.

	Whole food pattern	Processed food pattern
Other vegetables	**0.81**	−0.7
Leafy vegetables	**0.67**	−0.06
Cruciferous vegetables	**0.59**	0.05
Peas and dried legumes	**0.58**	0.17
Fruit	**0.56**	0.11
Whole grain	**0.59**	0.05
Fish	**0.49**	−0.29
Tomato	**0.41**	0.14
Egg	**0.37**	0.22
Low fat dairy	**0.34**	0.11
Hot drinks	**0.31**	0.10
Nuts	**0.30**	−0.12
Salad dressing	**0.30**	−0.04
Desserts/biscuits	0.19	**0.65**
Potatoes	0.10	**0.53**
Refined grains	−0.4	**0.53**
Fried foods	0.04	**0.52**
High fat dairy	0.07	**0.51**
Snacks	−0.07	**0.49**
Takeaway (high fat) ^1^	−0.05	**0.48**
Chocolate and sweets	0.20	**0.48**
Processed meat and fish	0.23	**0.43**
Sugar beverages	−0.08	**0.43**
Red meat	0.18	**0.43**

^1^ Included meat pie, sausage roll or other savoury pastries, pizza and hamburger.

### 2.3. Outcome: Cognition

Cognition at baseline was measured with the Cambridge Cognitive Examination (CAMCOG) [14], a valid measure of global cognitive performance [15]. The total score can range from zero to 105, with higher scores indicating a higher degree of cognitive function. CAMCOG contains eleven subscales that evaluate different cognitive functions: remote memory, recent memory, executive function, orientation, new learning, comprehension, expression, attention and calculation, praxis, abstract thinking and perception. Levels of cognitive functioning were defined by tertiles of performance of the total CAMCOG score and scores of individual subscales.

### 2.4. Covariates

Sociodemographic variables included age, gender, marital status (married, widowed, divorced/separated, single, defacto/cohabited), education (total years). Health behaviours included lifetime smoking (expressed in terms of pack-years, which was the mean number of cigarettes per day multiplied by the number of years smoking and divided by 20 [16]). Participants self reported if they did any physical activity, how many days they did physical activity and the average amount of physical activity they completed each day in minutes; there was no distinction made between moderate and vigorous physical activity. In a subset of 117 participants, the Human Activity Profile (HAP) was used to evaluate the participants’ physical activity [17]. The HAP is a self reported 96-item questionnaire, and items are ordered in increasing level of physical activity demand. Each item is marked as “still doing”, “have stopped doing” or “never did”. The Adjusted Activity Score was used in this study, which is the highest numbered item marked as “still doing” less the number of lower numbered items marked as “have stopped doing”. In order to determine if there was an association between the level of physical activity determined by the self reported and the HAP methods, a chi-square test of association was used. It was found that there was an association, χ^2^ (1, *n* = 80) = 10.824, *p *< 0.05); self reported physical activity was used in the final analysis (categorised as low: up to 40 min/day; medium 40–50 min/day, and high >60 min/day on average). Health status variables included use of antidepressants. 

### 2.5. Statistical Analysis

Statistical analysis was performed using the Statistical Package for the Social Sciences software version 17.0 (SPSS, Inc., Chicago, IL, USA) and Statistical Analysis Systems statistical software package version 9.1 (SAS Institute, Cary, NC, USA). Continuous variables were summarised as means ± SD and categorical data as proportions. All continuous variables were normally distributed. Sample characteristics were compared between three levels of dietary pattern scores using chi-square analysis and analysis of variance for categorical and continuous variables, respectively. Pearson’s correlations were used to analyse associations between physical activity cognition and dietary patterns. Logistic regression analysis examined the association between the dietary pattern score and cognitive functioning. Identified confounding factors included in the models were age, gender, education level, smoking pack years, physical activity and use of antidepressants. The self-reported number of years attending school was used as a measurement of level of education, and the self-reported number of minutes spent exercising per day was used as an indicator of physical activity. Levels of significance were set at 5% and all probability tests reported are two-tailed.

## 3. Results

### Participants

Ninety-four (38%) women and 155 (62%) men completed the baseline assessment with a mean ± SD age of 73.4 ± 5.6 years and CAMCOG total score of 89.8 ± 5.9. There were no apparent differences in demographic characteristics between the whole food and processed food patterns according to tertiles of dietary pattern score, except there was a higher proportion of women in the lowest tertile of processed food pattern, which suggests that women consumed less processed food than men (Table 2).

**Table 2:** Characteristics of study participants according to tertiles of dietary pattern score ^1^.

	Whole food dietary pattern				Processed food dietary pattern			
	(*n* = 249)				(*n* = 249)			
	Tertile 1 ^2^	Tertile 2 ^2^	Tertile 3 ^2^	*p* value	Tertile 1 ^2^	Tertile 2 ^2^	Tertile 3 ^2^	*p* value
	*n* = 82	*n* = 84	*n* = 83		*n* = 82	*n* = 84	*n* = 83	
Women/Men (%)	28/72	39/61	46/54	0.059 ^3^	62/38	31/69	20/80	<0.01 ^3^
Smoking status								
Current (%)	3.7	2.4	2.4	0.852 ^3^	3.7	3.6	1.2	0.555 ^3^
Cigarettes smoked, pack-years	32.2 ± 5.3	22.7 ± 3.5	18.8 ± 3.4	0.407 ^3^	23.5 ± 4.0	30.4 ± 5.8	21.9 ± 2.9	0.492
Age (years)	74.2 ± 5.7	73.0 ± 5.6	73.0 ± 5.5	0.279 ^4^	72.5 ± 5.5	73.4 ± 5.0	74.2 ± 6.1	0.145 ^4^
Total education (years)	11.6 ± 3.2	11.6 ± 2.9	12.5 ± 3.8	0.146 ^4^	12.3 ± 3.9	11.6 ± 2.9	11.9 ± 3.1	0.495 ^4^
Exercise (min/day)	67.1 ± 64.1	72.3 ± 61.2	54.8 ± 31.2	0.168 ^4^	57.8 ± 36.4	73.5 ± 69.8	62.3 ± 49.4	0.224 ^4^
CAMCOG total score	89.4 ± 6.6	90.5 ± 5.6	89.5 ± 5.5	0.432 ^4^	91.0 ± 5.0	89.3 ± 6.3	89.1 ± 6.2	0.080 ^4^

Abbreviations: CAMCOG, Cambridge Cognitive Examination; ^1^ Values are numbers, percentages and Mean ± SD; ^2^ Tertiles 1, 2 and 3 represent individuals in the lowest, intermediate and highest thirds of the dietary factor score, respectively; ^3^ χ^2^-test; ^4^ One way between groups ANOVA.

There was no association between the whole food pattern and the overall CAMCOG score (*r* = 0.007, *p* = 0.908); however, there was a negative correlation between the processed food pattern and the overall CAMCOG score (*r* = −0.153, *p* < 0.05). The logistic regression models adjusted for age, gender, education, smoking status, antidepressant use and physical activity (Table 3, model 3) showed that participants in the highest tertile of the processed food pattern score were more likely to have cognitive functioning in the lowest tertile as assessed with the CAMCOG subscale for executive function. The test for a linear trend was consistent with the impression that increasing levels of processed food in the diet are associated with an increased risk of being in the lowest tertile of cognition, although for model 3 (full adjustment), these were only statistically significant for executive function.

**Table 3:** Association between tertiles of processed food patterns on dietary questionnaires and the lowest tertile of cognitive functioning as assessed by CAMCOG and memory and executive function subscales in elderly participants with mild cognitive impairment (*n* = 249)^1^.

	Processed food pattern score							
	Tertile 1 (lowest)	Tertile 2			Tertile 3 (highest)			
**CAMCOG Total**	**OR**	**OR**	**95% CI**	***p* value** **^2^**	**OR**	**95% CI**	***p* value ^3^**	***p *of trend ^4^**
Model 1 ^5^	1.00	1.61	0.75, 3.43	0.220	2.07	0.96, 4.47	0.065	0.081
Model 2 ^6^	1.00	1.49	0.68, 3.27	0.322	1.73	0.78, 3.87	0.181	0.212
Model 3 ^7^	1.00	1.51	0.69, 3.33	0.303	1.70	0.76, 3.79	0.199	0.211
**CAMCOG subscales**								
Remote memory								
Model 1 ^5^	1.00	1.69	0.72, 3.96	0.228	2.63	1.11, 6.24	0.028	0.030
Model 2 ^6^	1.00	1.58	0.66, 3.82	0.306	2.32	0.94, 5.68	0.067	0.071
Model 3 ^7^	1.00	1.61	0.66, 3.91	0.292	2.41	0.97, 6.03	0.059	0.059
Executive function								
Model 1 ^5^	1.00	1.47	0.65, 3.32	0.357	2.36	1.04, 5.35	0.039	0.044
Model 2 ^6^	1.00	1.55	0.66, 3.64	0.317	2.62	1.11, 6.17	0.028	0.032
Model 3 ^7^	1.00	1.57	0.66, 3.71	0.095	2.55	1.08, 6.03	0.033	0.030

Abbreviations: CAMCOG, Cambridge Cognitive Examination; ^1^ All data are odds ratio and 95% confidence interval; ^2^ Tertile 1 *versus* tertile 2 (processed food); ^3^ Tertile 1 *versus* tertile 3 (processed food); ^4^ *p* value of the test for a linear trend for increasing tertile of food pattern score; ^5^ Adjusted for age, gender, education; ^6^ Adjusted for age, gender, education, smoking status, use of antidepressants; ^7^ Adjusted for age, gender, education, smoking status, use of antidepressants, physical activity.

## 4. Discussion

We examined associations between cognition and two dietary patterns: a whole food pattern (rich in vegetables, fruit, whole grain, fish, tomato egg and low fat dairy) and a processed food pattern (rich in desserts, biscuits, potatoes, refined grains, fried foods, high fat dairy, snacks, high fat takeaway, chocolate and sweets, processed meat and fish, sugar beverages and red meat). A processed food pattern was associated with poorer executive functioning; however, a whole food pattern was not associated with cognitive performance. 

There have been few studies published that have examined the relationship between dietary patterns, derived by factor analysis, and cognition. In a cross sectional study of 4693 middle aged participants from the Whitehall II study, principal component analysis derived two dietary patterns: a whole food and a processed food pattern. In agreement with our study, a dietary pattern consisting of processed foods was associated with higher odds of cognitive deficits [18]. In a smaller population based study, Gustaw-Rothenberg conducted factor analysis in 71 individuals with Alzheimer’s disease and 71 healthy controls [19]. This study also found that the group with Alzheimer’s disease was characterised by a processed dietary pattern, whereas the healthy controls had a healthy dietary pattern comprised of grains and vegetables. Nutrients that are common in a processed food pattern that have been associated with poorer cognition include refined sugars, cholesterol, alcohol and saturated fat [20]; foods in our processed food pattern that included these nutrients were desserts/biscuits, fried foods, high fat dairy, take away (high fat), chocolate and sweets and sugar beverages. 

Our finding that a whole food pattern was not associated with cognition conflicts with other published findings. Also from the Whitehall II study, a whole food dietary pattern was associated with lower odds of cognitive deficit [18], although this study consisted of middle aged healthy men and women, whereas in the present study we recruited older men and women with mild cognitive impairment. Gustaw-Rothenberg also found that healthy controls had a healthy dietary pattern consisting of grains and vegetables [19]. Wengreen *et al.* also found that a dietary pattern that included foods consistent with the Dietary Guidelines for Americans was associated with better cognitive scores [6]. However, it should be noted that this study used a hypothesis-driven, or a *Priori* approach to derive a food score, whereas in our study the whole food pattern was derived using principal components analysis. It is possible, that in the present study, we were unable to detect an association between cognition and the whole food dietary pattern due to a small sample size or in this specific cohort of older men and women with mild cognitive impairment. 

Several potential limitations of this study need to be highlighted. The cross-sectional design of this study prevents the detection of causal relationships between dietary patterns and cognition. We must also consider the possibility of reverse causality, such that cognitive decline may have already been present and this may have resulted in a diminished ability to purchase and prepare healthy foods, driving the link between the processed food pattern and cognitive decline. This study specifically recruited older adults with MCI; therefore, the findings may not be generalizable to a population of healthy older adults without MCI. Furthermore, we did not determine total energy intake and were unable to adjust for this in the logistic regression analysis.

## 5. Conclusion

In summary, in the group of older people with mild cognitive impairment, a higher intake of processed foods was associated with reduced memory and impaired executive function. 

## References

[1] (2005). Population Division of the Department of Economic and Social Affairs of the United Nations Secretariat. World Population Prospects: The 2004 Revision. Volume III Analytical Report.

[2] Winblad B., Palmer K., Kivipelto M., Jelic V., Fratiglioni L., Wahlund L.O., Nordberg A., Backman L., Albert M., Almkvist O. (2004). Mild cognitive impairment—beyond controversies, towards a consensus: Report of the International Working Group on Mild Cognitive Impairment. J. Intern. Med..

[3] Bennett D.A. (2003). Update on mild cognitive impairment. Curr. Neurol. Neurosci. Rep..

[4] Guehne U., Luck T., Busse A., Angermeyer M.C., Riedel-Heller S.G. (2007). Mortality in individuals with mild cognitive impairment. Results of the Leipzig Longitudinal Study of the Aged (LEILA75+). Neuroepidemiology.

[5] Donini L.M., de Felice M.R., Cannella C. (2007). Nutritional status determinants and cognition in the elderly. Arch. Gerontol. Geriatr..

[6] Wengreen H.J., Neilson C., Munger R., Corcoran C. (2009). Diet quality is associated with better cognitive test performance among aging men and women. J. Nutr..

[7] Vogel T., Brechat P.H., Leprêtre P.M., Kaltenbach G., Berthel M., Lonsdorfer J. (2009). Health benefits of physical activity in older patients: A review. Int. J. Clin. Pract..

[8] Lautenschlager N.T., Cox K., Kurz A.F. (2010). Physical activity and mild cognitive impairment and Alzheimer’s disease. Curr. Neurol. Neurosci. Rep..

[9] Welsh K.A., Butters N., Mohs R.C., Beekly D., Edland S., Fillenbaum G., Heyman A. (1994). The Consortium to Establish a Registry for Alzheimer’s Disease (CERAD). Part V. A normative study of the neuropsychological battery. Neurology.

[10] Morris J.C. (1993). The Clinical Dementia Rating (CDR): Current version and scoring rules. Neurology.

[11] Lines C.R., McCarroll K.A., Lipton R.B., Block G.A. (2003). Telephone screening for amnestic mild cognitive impairment. Neurology.

[12] Folstein M.F., Folstein S.E., McHugh P.R. (1975). “Mini-mental state”. A practical method for grading the cognitive state of patients for the clinician. J. Psychiatr. Res..

[13] Australian Bureau of Statistics (1998). 48010.0-National Nutrition Survey Users’ Guide.

[14] Roth M., Huppert F., Mountjoy C., Tym E. (1998). CAMDEX-R: The Cambridge Examination for Mental Disorders of the Elderly-Revised.

[15] Wouters H., van Gool W.A., Schmand B., Zwinderman A.H., Lindeboom R. (2010). Three sides of the same coin: Measuring global cognitive impairment with the MMSE, ADAS-cog and CAMCOG. Int. J. Geriatr. Psychiatry.

[16] Bernaards C.M., Twisk J.W., Snel J., van Mechelen W., Kemper H.C. (2001). Is calculating pack-years retrospectively a valid method to estimate life-time tobacco smoking? A comparison between prospectively calculated pack-years and retrospectively calculated pack-years. Addiction.

[17] Davidson M., de Morton N. (2007). A systematic review of the Human Activity Profile. Clin. Rehabil..

[18] Akbaraly T.N., Singh-Manoux A., Marmot M.G., Brunner E.J. (2009). Education attenuates the association between dietary patterns and cognition. Dement. Geriatr. Cogn. Disord..

[19] Gustaw-Rothenberg K. (2009). Dietary patterns associated with Alzheimer’s disease: Population based study. Int. J. Environ. Res. Public Health.

[20] Van Dyk K., Sano M. (2007). The impact of nutrition on cognition in the elderly. Neurochem. Res..

